# The Functional Analysis of Histone Acetyltransferase MOF in Tumorigenesis

**DOI:** 10.3390/ijms17010099

**Published:** 2016-01-14

**Authors:** Jiaming Su, Fei Wang, Yong Cai, Jingji Jin

**Affiliations:** 1 School of Life Sciences, Jilin University, Changchun 130012, China; xiaosuyoc@gmail.com (J.S.); fei@jlu.edu.cn (F.W.); caiyong62@jlu.edu.cn (Y.C.); 2 National Engineering Laboratory for AIDS Vaccine, Jilin University, Changchun 130012, China; 3 Key Laboratory for Molecular Enzymology and Engineering, the Ministry of Education, Jilin University, Changchun 130012, China

**Keywords:** MYST, MOF, histone acetyltransferase, H4K16ac, tumorigenesis

## Abstract

Changes in chromatin structure and heritably regulating the gene expression by epigenetic mechanisms, such as histone post-translational modification, are involved in most cellular biological processes. Thus, abnormal regulation of epigenetics is implicated in the occurrence of various diseases, including cancer. Human MOF (males absent on the first) is a member of the MYST (Moz-Ybf2/Sas3-Sas2-Tip60) family of histone acetyltransferases (HATs). As a catalytic subunit, MOF can form at least two distinct multiprotein complexes (MSL and NSL) in human cells. Both complexes can acetylate histone H4 at lysine 16 (H4K16); however, the NSL complex possesses broader substrate specificity and can also acetylate histone H4 at lysines 5 and 8 (H4K5 and H4K8), suggesting the complexity of the intracellular functions of MOF. Silencing of MOF in cells leads to genomic instability, inactivation of gene transcription, defective DNA damage repair and early embryonic lethality. Unbalanced MOF expression and its corresponding acetylation of H4K16 have been found in certain primary cancer tissues, including breast cancer, medulloblastoma, ovarian cancer, renal cell carcinoma, colorectal carcinoma, gastric cancer, as well as non-small cell lung cancer. In this review, we provide a brief overview of MOF and its corresponding histone acetylation, introduce recent research findings that link MOF functions to tumorigenesis and speculate on the potential role that may be relevant to tumorigenic pathways.

## 1. Introduction

The nucleosome as the basic repeating unit of chromatin is composed of chromosomal DNA and a histone octamer, which contains two copies each of the core histones H2A, H2B, H3 and H4. With linker DNA together, numerous nucleosomes fold into compact higher order chromatin structures in the nucleus of eukaryotic cells [1]. The compact status of the chromatin structure determines the accessibility of DNA. Loosened chromatin structures will alter their shape, freeing DNA, allowing the binding of transcription factors and other sequence-specific regulators [2]. These changes in chromatin structure regulate many critical DNA transactions, including transcription, replication, recombination and repair. Alterations in chromatin structure can be produced by two major mechanisms: (1) post-translational modifications of the N-terminal tails of histones by chromatin-modifying enzymes can recruit specific effector proteins to chromatin, thus affecting the chromatin structure; and (2) ATP-dependent chromatin remodeling complexes can alter the chromatin structure by affecting the interaction between DNA and histones [3,4,5]. The various modifications of histone N-terminal tails, such as acetylation, methylation, ubiquitination and phosphorylation, via heritably regulating the gene expression, are involved in most cellular biological processes. For instance, imbalanced histone modifications affect genome integrity and/or chromosome segregation [6]. Current evidence has indicated that disproportionate global histone modifications in cells may play a key role in initiating events in some forms of cancer by altering gene expressions, including aberrant regulation of oncogenes and/or tumor suppressors [7,8]. Histone acetylation is dynamically controlled by HATs and histone deacetylases (HDACs) [9]. Human MOF (also known as human MYST1) is a member of the MYST family of HATs. It has been verified that depletion of MOF can influence a wide range of intracellular biological functions, including chromatin stability, cell cycle, gene transcription, DNA damage repair and early embryonic development [10,11,12,13]. In this review, we focus on the MOF and its corresponding histone acetylation, summarize the current understanding of MOF functions on tumorigenesis and speculate on the potential role that may be relevant to tumorigenic pathways.

## 2. MOF Belongs to the MYST Family of HATs

Global histone acetylation in cells controlled by HATs and HDACs plays an important role in regulation of chromatin structure and function [9,14]. Based on sequence homology, as well as shared structural features and functional roles, HATs can be divided into two different classes: the GCN5-related *N*-acetyltransferases (GNATs) family, including GCN5 and p300/CBP-associating factor (PCAF), are generally characterized by the presence of a bromodomain, and they can acetylate lysine residues on histone H2B, H3 and H4 [15,16]; however, the MYST family, which is characterized by a highly conserved MYST domain, is composed of an acetyl-CoA-binding motif and a zinc finger [17,18]. Except for the conserved MYST domain, some MYST family members also possess additional structural features, such as chromodomain (MOF, Esa1 and Tip60), plant homeodomain-linked zinc fingers (Moz and MORF) and other domains that bind specifically to modified histones [18].

Males absent on the first (MOF) was originally identified as one of the components of the dosage compensation in *Drosophila* [19]. Based on experimental results and sequence analysis, MOF is a putative HAT gene, which is related to the Tip60 and Moz human genes and to the *SAS* genes of yeast. Therefore, MOF was classified into the MYST family [20]. In *Drosophila*, MOF-containing dosage compensation or male-specific lethal (MSL) complex plays a key role in equalizing X-linked gene expression between male and female flies. Research evidences indicate that MSL complexes directly bind to the X chromosome of *Drosophila* males and specifically acetylates histone H4K16, thereby leading to hyper-transcription of the X chromosome in males, suggesting the roles of the MSL complex in dosage compensation [20,21,22,23,24].

**Figure 1:**
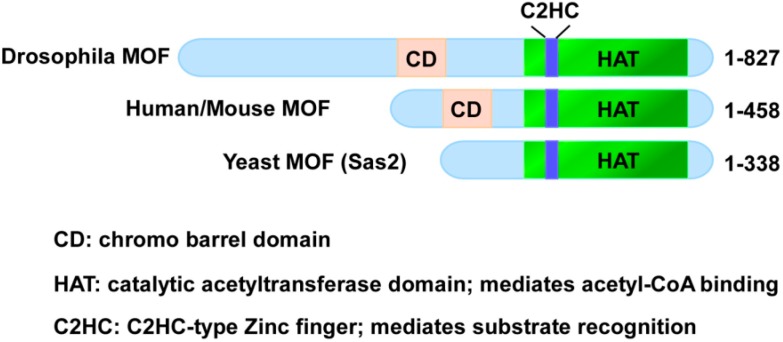
Functional domains of the MOF protein.

The human ortholog of *Drosophila* MOF exhibits significant similarity to the *Drosophila* MOF containing an acetyl-CoA-binding site, a chromodomain, which binds histones, and a C2HC-type zinc finger [25]. The functional domain of MOF between different species is shown in Figure 1. MOF has the same substrate specificity and is highly conserved from fly to human [13]. However, whether the human MSL complex is also involved in the mechanism of dosage compensation in human cells is still unclear. However, recent evidence from mouse embryonic stem cells (ESCs) indicates that specific histone marks, including H4K16 acetylation (H4K16ac) and histone variant H2AZ, are enriched at the active mouse X-linked promoters. ChIP-chip analyses using genome tiling arrays confirmed that the occupancy of MOF and RNA polymerase II (PolII-S5p) is enriched at expressed X-linked genes in female ESCs, and knockdown MOF decreased PolII-S5p occupancy, suggesting that upregulation of mouse X chromosome is associated with enhanced transcription initiation and MOF-mediated H4K16 acetylation [26].

## 3. MOF Forms Two Distinct Multiprotein Complexes in Cells

The MSL complex or the dosage compensation complex (DCC) was first characterized in the study of the dosage compensation of *Drosophila* males. The components of the MSL complex include MSL1, MSL2, MSL3, MLE, MOF and at least one of the two noncoding roX RNAs (roX1 and roX2), which associates with the male X chromosome [27,28]. Later research found that direct interaction of MOF with MSL1 and MSL3 is more important than roX RNA for recruiting the complex to chromatin and dramatically increasing the HAT activity of MOF [29]. Biochemical purification approaches confirmed that conserved multiprotein complexes exist between *Drosophila* and mammals. Human MOF (hMOF) can also form the human MSL (hMSL) complex, which contains highly conserved proteins through evolution, such as hMSL1, hMSL2 and hMSL3. In addition, hMSL complex has the same substrate specificity and is specifically responsible for histone H4K16ac in human cells [30,31].

In 2005, Dou *et al.* [32] purified a multiprotein complex from Flag-WDR5 (WD repeat domain 5) stably expressing HeLa nuclear extract. In addition to the subunits of the MLL (mixed-lineage leukemia) complex, MOF is stably associated in cells with WDR5 and other proteins other than subunits of the MSL complex, such as PHF20 (plant homeodomain-linked finger-containing protein 20), HCF1 (host cell factor 1) and MCRS 2 (microspherule protein 2). Interestingly, Flag-WDR5 complexes possess both histone methyltransferase (HMT) and HAT activities, suggesting the existence of additional MOF-containing HAT complexes. Consistent with this idea, using biochemical purifications and mass spectroscopy, Akhtar and co-workers confirmed that MOF is a shared subunit of two distinct multi-protein complexes in mammals and *Drosophila*.

Except for the MSL complex, MOF is considered to be a component of a second HAT complex, which is designed as the non-specific-lethal (NSL) complex [29]. Subsequently, proteomics analyses of complexes purified through multiple candidate subunits from human cells revealed that the NSL complex is composed of nine subunits: catalytic subunit-MOF, NSL1 (KIAA1267), NSL2 (FLJ20436), NSL3 (FLJ10081), MCRS1 (microspherule protein 1), WDR5, *O*-linked *N*-acetylglucosamine transferase, isoform 1 (OGT1), HCF1 and PHF20. *In vitro* HAT assays verified that the NSL complex presents broader substrate specificity and can acetylate nucleosomal histone H4 on lysines 5, 8 and 16 [33]. Additionally, NSL-associated MOF appears to be involved in more global regulation of transcription [34,35,36].

**Figure 2:**
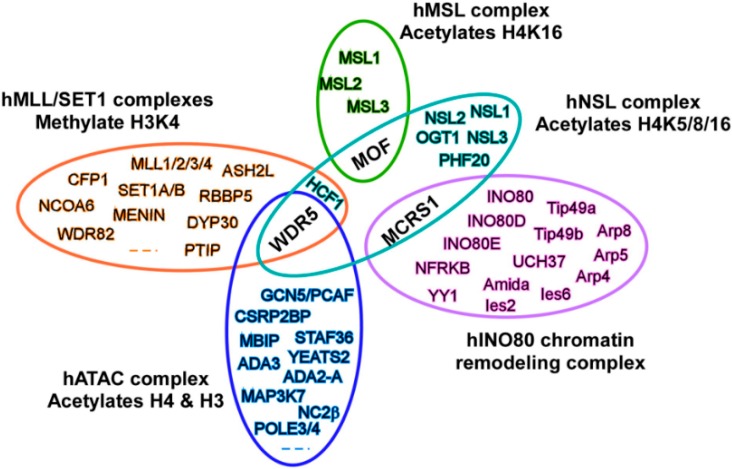
The NSL complex shares subunits with other chromatin-regulating complexes. hMLL, human mixed-lineage leukemia; SET, set-domain containing; INO80, INOsitol-requiring 80; NFRKB, nuclear factor related to kappaB protein; YY1, Yin Yang 1; GCN, general control of amino acid synthesis; MBIP, MAP3K12 binding inhibitory protein 1; ADA, transcriptional adaptor; STAF, YEATS2, YEATS domain-containing 2; NC2β, down-regulator of transcription 1; CSRP2BP, CSRP2 binding protein; RBBP, retinoblastoma binding protein; DYP30, protein dpy-30 homolog; PTIP, PAX interacting (with transcription-activation domain) protein 1; WDR, WD repeat domain.

It is noteworthy that the NSL complex shares subunits with other chromatin-regulating complexes. As shown in Figure 2, WDR5 protein is shared by at least three different complexes, including the NSL complex, the MLL-SET (set-domain containing) histone H3K4 methyltransferase complex [37] and the ATAC histone acetyltransferase complex [38]. Meanwhile, the MCRS1 protein is contained in two different complexes, the NSL and the INO80 chromatin remodeling complexes [39]. Given the cross-shared subunits between those complexes, it is easy to imagine functional links between different chromatin regulatory complexes. It has been reported that H4K16ac and H4K20me3 modified by MOF and SUV420H2, respectively, can antagonistically control gene expression through regulating Pol II promoter-proximal pausing [40]. Coordination between NSL-mediated H4K16ac and MLL/SET complex-mediated histone H3K4 di-methylation (H3K4me2) has also been confirmed by a series of biochemical experiments. The NSL complex can promote H3K4me2 by MLL/SET complexes via an acetylation-dependent mechanism. Chromatin immunoprecipitation (ChIP) assays further clarified that a set of genes, including ANKRD2, a muscle ankyrin repeat protein, are co-regulated by NSL and MLL/SET complexes [41]. In addition, H4K16ac by the NSL complex can promote c-Jun target gene transcription, suggesting that the NSL complex might be a c-Jun co-activator in c-Jun-regulated gene expression [42]. We believe that more complex MOF-related functions exist in cells, since some enzymes, such as SIRT1 and HDAC3, are also involved in acetylating histone H4K16 in cells [43,44,45].

## 4. MOF Plays a Critical Role in Cells

It has been known that histone acetylation is required for many intracellular biological processes, such as gene regulation, genome maintenance and organism development. As a key enzyme for mainly acetylating histone H4K16, MOF-containing complexes have been implicated in various essential cellular functions with obvious links to cancer [46]. Except for the global reduction of histone H4K16ac, depletion of MOF in mammal cells can result in abnormal gene transcription, especially causing abnormal expression of certain tumor suppressor genes or oncogenes [11,12,47], suggesting the critical roles of MOF in tumorigenesis.

### 4.1. MOF in Gene Transcriptional Regulation

Modification of N-terminal tails of histones plays a central role in regulating gene activation and silencing. Histone H4 can be acetylated at several lysine residues on the N-terminal tail, including K5, K8, K12 and K16 in all eukaryotes. Acetylation of lysine residues on histone H4 loosens the chromatin structure by neutralizing their positive charge and facilitates the binding of various factors, such as transcriptional factors and co-activators [1,23]. For example, high levels of H4K16ac by Sas2 (something about silencing), the yeast ortholog of MOF, inhibit binding of the telomeric gene silencing-associated protein Sir3 [48], while in *Drosophila*, H4K16ac by MOF is related to the binding of the chromatin compaction-related protein ISWI ATPase [49]. Both *in vitro* and *in vivo* assays have shown that MOF-mediated H4K16ac results in increased transcription and a two-fold transcriptional regulation of male X chromosomal genes during dosage compensation in *Drosophila* [20,26,50]. Genome-wide analysis in *Drosophila* reveals that MOF is distributed at both the promoters and 3′ ends of genes of male X chromosome [10], and this distribution is closely associated with two distinct MOF-containing complexes, the MSL and NSL complexes. Enrichment of the MSL complex is also towards the 3′ end of genes on the male X chromosome. In contrast to the MSL complex, theMBD-R2/MOF complex (MBD-R2 is a component of the NSL complex) is enriched towards the 5′ end of genes on all female and male autosomal genes and peaks around the transcriptional start site (TSS) [51], suggesting that NSL-mediated MOF is not only a transcriptional regulator, but it is also implicated in gene transcriptional regulation independent of the DCC in a wider range [34]. The subsequent studies have confirmed that the majority of the NSL complex-bound targets in *Drosophila* are housekeeping genes, but only a defined subset of genes are activated by the NSL complex [35,36]. Further evidence of MOF involvement in transcriptional regulation has been uncovered from recent studies. H4K16ac by human MOF regulates the outcome of autophagy by controlling the autophagy-related genes [52]. In proliferating cells, MOF regulates the expression of cell cycle progression-related genes. Furthermore, NSL-mediated MOF can directly bind to genes that are required for podocyte maintenance [53]. In addition, DNA microarray analyses show a total of 346 genes to be differentially expressed by >2-fold between the hMOF siRNA knockdown and non-targeting siRNA-treated HeLa cells. One-hundred ninety-five of the 346 genes, including HCP5 (human leukocyte antigen complex P5), are downregulated [54]. Polysialic acid (PSA) is an unusual post-translational modification that occurs on neural cell adhesion molecules. Thus, polysialylation of cell adhesion molecules is a key step in neuronal rewiring and allows normalization of food intake. Brenachot and colleagues [55] found that upon high fat diet, MOF is rapidly recruited on the *St8sia4* polysialyltransferase-encoding gene and activates hypothalamic polysialylation to prevent diet-induced obesity in mice, suggesting the important role of MOF in regulating the energy balance.

It is necessary to remember that non-histone proteins, such as p53 and NF-E2-related factor 1 (Nrf2), can also be the substrates of MOF. For example, acetylation of p53 at K120 by MOF affects its binding to DNA and leads to increased transcription of pro-apoptotic genes, like PUMA (p53 upregulated modulator of apoptosis) andBAX (bcl-2 associated X protein) [56]. On the other hand, MOF is able to bind and acetylate Nrf2 at K588, thereby facilitating its nuclear translocation and increasing transcription of Nrf2 target genes [57]. However, acetylation of TIP5 (a subunit of the human NoRC chromatin-remodeling complex) at K633 by MOF is required for NoRC-mediated rDNA silencing [58]. In *Drosophila*, a component of MSL complex-MSL3 can be acetylated by MOF at K116, therefore affecting the interaction between MSL3 and RNA and leading to the fine-tuning of dosage compensation [59]. Autoacetylation of MOF at K274 has been reported by several groups. K274 is a highly conserved lysine residue in the C2HC zinc-finger domain of MOF [60,61]. Various mutants of K274 of the C2HC domain or blocking the C2HC domain lead to lack of MOF enzyme activity, suggesting that acetylation of K274 is critical for MOF substrate specificity and HAT activity [60,61,62].

### 4.2. Functions of MOF in DNA Damage Repair

DNA damage and mutations in oncogenes or tumor suppressor genes often occur in carcinogenesis. Both Saccharomyces cerevisiae NuA4 and its mammalian homolog Tip60 are members of the MYST family of HATs. Mutation in HAT NuA4 leads to increased sensitivity to DNA-damaging reagents in yeast [63], while in higher eukaryotes, HAT Tip60 regulates the DNA damage repair process through acetylation of both H2A and H2AX [64,65]. Within the MYST family, MOF may have a similar function. As expected, experimental results clarify that acetylation of H4K16 by MOF plays a critical role during multiple stages in the DNA damage response (DDR) and DNA damage repair pathways. Mutation Drosophila MOF or depletion MOF in SL-2 cells not only reduces post-irradiation survival, but it also results in a defective mitotic checkpoint and a defective p53 response post-irradiation, suggesting the involvement of MOF in DDR [66]. Furthermore, depletion of MOF blocks repair of DNA double-strand breaks (DSBs) by both the nonhomologous end-joining (NHEJ) and homologous recombination (HR) pathways [12]. Research evidence has shown that chromatin modifiers facilitate the accumulation and function of DNA repair proteins at the damaged loci [67]. Involvement of human MOF in ATM (ataxia-telangiectasia-mutated) was first mentioned by Gupta and colleagues [68]. In ionizing radiation (IR)-treated cells, hyper-acetylation of H4K16 by MOF was observed. Interestingly, blocking the IR-induced hyper-acetylation of H4K16 led to decreased ATM autophosphorylation and low-ATM kinase activity, thereby resulting in decreased phosphorylation of downstream effectors of ATM, suggesting the function of MOF in regulating ATM [68]. Afterwards, ATM-dependent MOF phosphorylation at the T392 site (p-T392-MOF) was observed during DSBs of DNA, and p-T392-MOF was shown to colocalize with γ-H2AX, ATM and p53BP1 foci; this indicates that ATM-mediated p-T392-MOF might modulate p53BP1 function to facilitate the subsequent recruitment of HR repair proteins, such as BRCA1 and MDC1 [69,70]. Taken together, MOF plays an important role in the DNA DSB repair pathway. Additional evidence indicates that MOF functions in DDR through interacting with the DNA-dependent protein kinase catalytic subunit (DNA-PKcs), showing the roles of MOF in the NHEJ pathway [12].

### 4.3. Functions of MOF in ESCs

**Figure 3:**
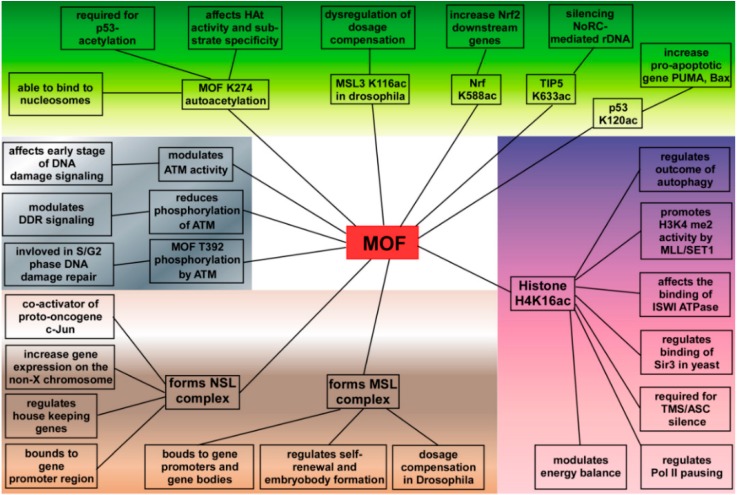
Functional regulation of MOF in cells. TMS, Target of methylation-mediated silencing, also known as ASC. Green: non-histone proteins are acetylated by MOF; Purple: pathways associated with MOF-mediated H4K16ac; Brown: functions associated with MOF-containing complexes; Gray: MOF is implicated in DNA damage repair.

Embryonic stem cells (ESCs) are unique cells that can self-renew and differentiate into many cell types. Several transcription factors, such as Oct4, Nanog and Sox2, and cell surface proteins have been considered to ensure the suppression of genes leading to the differentiation and maintenance of pluripotency. Histone post-translational modifications play an important role in the regulation of ESCs’ self-renewal and pluripotency. Research evidence has revealed that HAT Gcn5, p300/CBP [71,72] and some members of the MYST family (MOF, Tip60/P400) are required for the development and self-renewal of stem cell populations [73,74]. Li and colleagues [75] found that the expression of MOF and its corresponding modification H4K16ac are significantly higher in ESCs than in mouse embryonic fibroblasts (MEFs). However, the levels of MOF in both mRNA and proteins are decreased in differentiated ESCs by retinoic acid and in embryoid bodies [75]. Simultaneously, the expression of key pluripotency genes, such as Oct4, Nanog and Sox2, also decreased. In contrast, the expression of Oct4, Nanog and other conserved common targets are decreased in the absence of MOF ESCs, suggesting the important role of MOF in maintaining ESC function [76]. Furthermore, MOF-deficient embryos not only failed to specifically acetylate H4K16, but they also failed to develop beyond the expanded blastocyst stage and died at implantation *in vivo*, demonstrating that MOF might be required specifically for the maintenance of H4K16ac in early embryos [74]. In later studies, overlapping and unique roles of the MOF-containing MSL and NSL complexes in regulating pluripotency in ESCs have been reported. For example, both complexes bind to specific and common sets of expressed genes; however, the NSL complex binds more in the promoter region, whereas the MSL complex binds in gene bodies [77]. In addition, the potential regulation of MOF-containing MSL and NSL complexes in ESCs and neuronal progenitors has been studied. MSL and NSL complexes revealed the distinct dynamics during differentiation from ESCs to neuronal progenitor cells (NPCs). Compared to the housekeeping functions of the NSL complex, the MSL complex predominantly performs more specialized roles. For example, although both complexes regulate gene expression by targeting promoters and transcription start site (TSS) distal enhancers, the MSL complex specifically regulates *Tsix*, the major repressor of Xist lncRNA. However, the NSL complex regulates Xist expression by maintaining pluripotency in a *Tsix*-independent manner [78]. Recent data also exhibit that *Drosophila* MOF may control checkpoint protein 2 (Chk2) and thereby regulates genomic stability during early embryogenesis [79]. A summary of the different known functions of MOF in cells is shown in Figure 3.

## 5. Roles of MOF in Tumorigenesis

It has been proven that epigenetic mechanisms through heritably regulating the organization and expression of genetic information are involved in almost all biological processes. Because of this, abnormal regulation of epigenetics is implicated in the occurrence of various diseases, including cancer [8,80]. Histone N-terminal modifications through a variety of modifying enzymes can directly affect gene expression by altering the chromatin structure. The different histone modifications result in either transcriptionally-active or -repressive marks. For example, specific lysine methylation of H3K4, H3K36 and H3K79 is often associated with active genes in euchromatin, whereas methylation of H3K9, H3K27 and H4K20 is associated with heterochromatin [81]. Conversely, acetylation of lysine residues on histones is generally associated with transcriptional activation [82,83]. Recent findings on the mechanism by which HATs or HDACs contribute to tumorigenesis and metastasis have been intensively studied in several types of cancer, as described below.

### 5.1. Histone Acetylation Modifiers in Cancer

Global histone lysine acetylation levels in cells are controlled by HATs and HDACs. Eighteen human HDAC family members have been identified and are grouped into four classes. Classes I (including HDAC1/2/3/8), IIa (including HDAC4/5/7/9), IIb (including HDAC6/10) and IV (HDAC11) are Zn^2+^-dependent metalloproteins, while the nicotinamide adenine dinucleotide (NAD^+^)-dependent enzymes (also known as sirtuins, SIRTs) are classified as the Class III family of HDACs (including SIRT1-7) [84]. Altered expression of HATs and HDACs has been observed in a variety of cancers. For example, low expression of HAT TIP60 has been found in gastric cancer, and the reduction of TIP60 shows a significant correlation with lymph node metastasis [85]. On the other hand, HDAC SIRT1 (sirtuin 1) plays a tumor-suppressive role in gastric cancer development by inhibiting NF-KB signaling [86]. Furthermore, overexpressed HDAC1, 2, 3, 4 and 6 are observed in prostate, cervical, renal cell or/and breast carcinomas, respectively [80,87,88]. Aberrant lysine acetylation by HATs has been implicated in initiating events in some cancers. The HATs, such as p300, CBP, MOZ and MORF, interact with Runx1 (or AML1), which is one of the most frequent targets of chromosomal translocations in leukemia. The genes for those HATs are rearranged in recurrent leukemia-associated chromosomal abnormalities, suggesting that perturbation of the intracellular acetylation program may be implicated in leukemogenesis [89]. Similarly, numerous examples of HATs involved in recurrent chromosomal translocations (e.g., MLL-CBP) or coding mutations (e.g., p300/CBP) are found in a broad range of solid and hematological malignancies [90,91,92,93]. It is worth mentioning that HATs can also acetylate non-histone proteins, including many oncogenes and tumor suppressor genes, such as MYC (c-Myc) and p53, indicating the importance of acetylation in both histone and non-histone proteins in cancer development [32,94,95]. High mobility group box (HMGB) proteins, the second most abundant chromosomal proteins, can also be acetylated by certain HATs. For instance, HMGB1 and HMGB2 proteins are the substrates for CBP in *in vitro* HAT assays [96]. Acetylated HMGB1 protein modulates its interaction with supercoiled DNA [97]. During apoptosis, low acetylation of histones may cause a hypercondensation of chromatin and irreversible HMGB1 binding [98]. Moreover, just like Tip60 and MOF, HMGB proteins are also implicated in various essential cellular functions, such as DNA transcription, replication, repair and recombination, with obvious links to cancer [99,100,101,102]. In fact, abnormal expression of HMGB proteins in ovarian and prostate cancer tissues has been observed [100]. Thus, given that HMGB proteins are part of chromosomal proteins and the substrates for HATs, the interaction between HTAs and HMGB proteins needs to be have closer attention paid in the future to clarify the mechanism of histone acetylation modifiers in cancer.

### 5.2. MOF Expression in Cancer

In mammalian cells, MOF is mainly responsible for acetylation of histone H4K16; in other words, the status of H4K16ac is closely related to MOF expression level. Abnormal expression of MOF and its corresponding modification H4K16ac have been found in different types of tumor tissues and cancer cells. In particular, loss of H4K16ac is now considered a common hallmark in human cancer [103]. The examination of the MOF expression in breast cancer and medulloblastoma clearly shows that MOF is frequently downregulated (>2-fold) in 41% of patients with primary breast carcinoma and in 79% of patients with medulloblastoma. Furthermore, the MOF protein expression is tightly correlated with H4K16ac in all tested samples [104]. In addition, medulloblastoma patients with low MOF expression show a significantly worse overall survival [104]. Subsequent research supported these results. Downregulation of MOF gene expression in certain primary cancers, including renal cell carcinoma (RCC) [105], ovarian cancer [54,106], gastric cancer [107,108], hepatocellular carcinoma [109] and colorectal cancer [108], was clarified. As expected, the MOF protein expression tightly correlated with H4K16ac in all tested tumors. Importantly, in patients with RCC, low expression of MOF is connected to clear cell RCC and tissues with T1 tumor status [108]. However, in patients with colorectal cancer, the patterns of MOF expression were mainly associated with lymph node metastasis and tumor stage [108]. It is noteworthy that in patients with gastric cancer, significant reduction (>2-fold) of MOF expression (35% of patients) had already appeared in adjacent tissues in which the cells were pathologically normal [108], suggesting that MOF may be related to the initial process of tumorigenesis. Notably, in hepatocellular and gastric carcinoma, patients with high levels of MOF had a longer time of survival than those with low levels of MOF [107,109]. Relatively, a considerable portion of tissues with primary non-small cell lung cancer (NSCLC) showed high expression of MOF in 33%, 34% and 50% from different research groups, respectively [57,110,111]. No matter how the MOF expression changes (downregulated or upregulated), there is no doubt that MOF is involved in the development of a variety of human cancers. Considering that the global H4K16ac in cells is balanced by HATs and HDACs, we could not rule out the regulation of other enzymes on H4K16ac. Recent evidence has verified that H4K16ac in cells can also be regulated by some HDACs, such as SIRT1 and HDAC2 [112,113,114]. In gastric cancer cells, low-expression of MOF and overexpression of HDAC4 were detected. However, the global H4K16ac in gastric cancer cells is increased by overexpressing MOF, but not by knocking down HDAC4, suggesting that the expression of MOF might be responsible for the global histone H4K16ac in gastric carcinoma [107]. A summary of the aberrant expression of MOF in cancer tissues/cells is shown in Table 1.

### 5.3. Regulation of MOF in Cancer

The association between the DNA and the histones or between different nucleosomes can be loosened by histone acetylation. Thus, hyper-acetylation results in opening up the chromatin and increasing the accessibility of nucleosomal DNA to transcriptional factors and other co-activators [13,115]. In *Caenorhabditis elegans*, H4K16ac regulates the formation of the higher structure of chromatin, including condensed 30-nm chromatin fiber and cross-fiber interactions [116], suggesting the critical role of H4K16ac in regulating gene transcription and cellular function. Research data have indicated that the reduction of global H4K16ac in cancer is tightly connected to MOF. Thus, it is easy to speculate that MOF and its corresponding H4K16ac may be involved in the occurrence of cancer by regulating certain oncogenes or tumor suppressor genes. Consistent with this idea, Kapoor-Vazirani and colleagues found that TMS1/ASC, a pro-apoptotic gene that undergoes epigenetic silencing in human cancers, was regulated by MOF-dependent H4K16ac in breast cancer cells [117]. In mice, loss of MOF and H4K16ac increased genomic instability by affecting cell proliferation and cell survival, indicating that the MOF is essential for embryogenesis and oncogenesis in mammalian cells [11]. In other cases, MOF may suppress hepatocellular carcinoma growth through regulating SIRT6 expression [100]. Microarray analysis and ChIP assay suggest that MOF modulates tumorigenesis in lung cancer and that it promotes S phase entry by regulating H4K16ac [111].

### 5.4. Epigenetic Therapies Targeting Histone Acetylation in Cancer

Histone acetylation is a well-characterized epigenetic modification controlled by HATs and HDACs. It is now widely accepted that the pathways of histone acetylation play an essential role in tumorigenesis. Therefore, efforts have been made to understand the role of global changes of histone acetylation in the tumorigenesis of various cancers and to find the drugs or small molecules that can reverse the acetylation levels to normal in cancer cells. In fact, the high-expression of HDACs in cancer has attracted the attention of the scientific community. Therefore, various synthetic small molecules, such as HDAC inhibitors (HDACi), have been used for anti-tumor testing. Increasing evidence has verified that HDACi can restore certain aberrantly-silenced genes, as well as induce growth arrest, differentiation and apoptosis of tumor cells [118,119,120,121]. So far, three HDACi, including Vorinostat (suberoylanilide hydroxamic acid, SAHA; Zolina), Romidepsin (Istodax, FK228, FR901228, depsipeptide) and Belinostat (Beleodaq, PXD-101, chemical name: (2*E*)-*N*-Hydroxy-3-[3-(phenylsulfamoyl)phenyl]prop-2-enamide), have been approved by the FDA and are in clinical use for the treatment of cutaneous T-cell lymphoma (SAHA, Istodax) and peripheral T-cell lymphoma (Beleodaq and Istodax) [122,123,124]. In addition, some HDAC inhibitors are at different stages of clinical development for the treatment of many types of cancer [82,125]. For example, hydroxamic acid-based HDACi, such as pracinostat (SB939, (*E*)-3-(2-butyl-1-(2-(diethylamino)ethyl)-1*H*-benzo[d]imidazol-5-yl)-*N*-hydroxyacrylamide), abexinostat (PCI-24781,: 3-((dimethylamino)methyl)-*N*-(2-(4-(hydroxycarbamoyl)phenoxy)ethyl)benzofuran-2-carboxamide), givinostat (ITF2357, 6-((diethylamino)methyl)naphthalen-2-yl) methyl 4-(hydroxycarbamoyl)phenylcarbamate hydrochloride hydrate)), resminostat (RAS2410, 2-Propenamide, 3-[1-[[4-[(dimethylamino)methyl]phenyl]sulfonyl]-1*H*-pyrrol-3-yl]-*N*-hydroxy-, (2*E*)-), quisinostat (JNJ-26481585, *N*-hydroxy-2-(4-(((1-methyl-1*H*-indol-3-yl)methylamino)methyl)piperidin-1-yl)pyrimidine-5-carboxamide dihydrochloride), CUDC-101 (7-(4-(3-ethynylphenylamino)-7-methoxyquinazolin-6-yloxy)-*N*-hydroxyheptanamide) and panobinostat (LBH589, (*E*)-*N*-hydroxy-3-(4-((2-(2-methyl-1H-indol-3-yl)ethylamino)methyl)phenyl)acrylamide), are in different stages of clinical studies [126]. It is important to remember that the decreased global histone acetylation in cancer may also be due to the low level of HATs and their corresponding enzyme activities. Reduction of MOF expression and its corresponding modification H4K16ac in various types of tumors have been clarified by experimental data. Research evidence has also suggested that the anti-cancer mechanism of some HDACi (trichostatin A and valproic acid) may be caused by increasing the acetylation levels of certain lysines on histones. Hajji and colleagues [43] found that MOF is required for the HDACi-mediated increase in H4K16ac and the cell-death-promoting effect. Furthermore, overexpression of MOF can suppress hepatocellular carcinoma growth *in vitro* and *in vivo* [108]. Thus, efforts to screen and discover compounds that are able to reverse the intracellular acetylation levels to normal by HATs, such as MOF in tumor cells, might become the next research goal.

**Table 1:** Changes in H4K16 acetylation and histone modifiers occurring in different cancers.

Cancer Type	Samples	Method	Modifiers	Results	H4K16ac	Reference
**Breast cancer**	Tissue (98)	Gene profiling	MOF	Downregulated (40%)	----	[104]
	Tissue (298)	IHC	MOF	Reduced or undetectable (18%)	H4K16ac ↓	[104]
	Cell lines	RT-PCR, WB	MOF/SUV420H2	Regulate Pol II pausing	----	[40]
	Cell lines	RT-PCR, WB	MOF	Silencing *TMS1* gene	H4K16ac ↓	[117]
**RCC**	Tissue (47)	RT-qPCR	MOF	Reduced mRNA (74%)	H4K16ac ↓	[108]
	Tissue (21)	RT-PCR, WB, IHC	MOF	Reduced mRNA/protein (91%)	H4K16ac ↓	[105]
**Medulloblastoma**	Tissue (14)	RT-PCR	MOF	mRNA downregulated (79%)	----	[104]
	Tissue (180)	IHC	MOF	Reduced or undetectable (40%)	H4K16ac ↓	[104]
**Gastric cancer**	Tissue (16)	RT-qPCR	MOF	mRNA downregulated (94%)	----	[108]
	Tissue (52)	RT-qPCR	MOF	mRNA downregulated (81%)	----	[107]
	Cell lines	RT-qPCR, WB, IF	MOF/HDAC4	Low MOF/high HDAC4	H4K16ac ↓	[107]
**Colorectal cancer**	Tissue (44)	RT-qPCR, IHC	MOF	Reduced mRNA (57%)/protein	H4K16ac ↓	[108]
**Ovarian cancer**	Tissue (57)	RT-qPCR, WB, IHC	MOF	Reduced mRNA/protein (65%)	H4K16ac ↓	[54]
	Tissue (30)	RT-PCR, WB	MOF	Reduced mRNA/protein	H4K16ac ↓	[106]
**Hepatocellular**	Tissue (70)	RT-qPCR, WB, IHC	MOF	Reduced mRNA/protein	----	[109]
	Cell lines	RT-qPCR	MOF	Reduced mRNA	----	[109]
**NSCLC**	Tissue (43)	IHC	MOF	Increase protein (14/43)	H4K16ac ↑	[111]
	Tissue (54)	RT-PCR, IHC	MOF	Increased mRNA/protein (>34)	----	[57]
	Tissue (20)	RT-PCR	MOF	Increased mRNA (50%)	----	[110]

RCC: Renal cell carcinoma; NSCLC: non-small cell lung carcinoma; gene profiling: gene expression profiling; IHC: immunohistochemistry; RT-PCR: reverse transcription PCR; qPCR: real-time quantitative PCR; WB: Western blot; IF: Immunofluorescence:, ↓: low level of H4K16ac; ↑: high level of H4K16ac.

## 6. Conclusions and Perspectives

**Figure 4:**
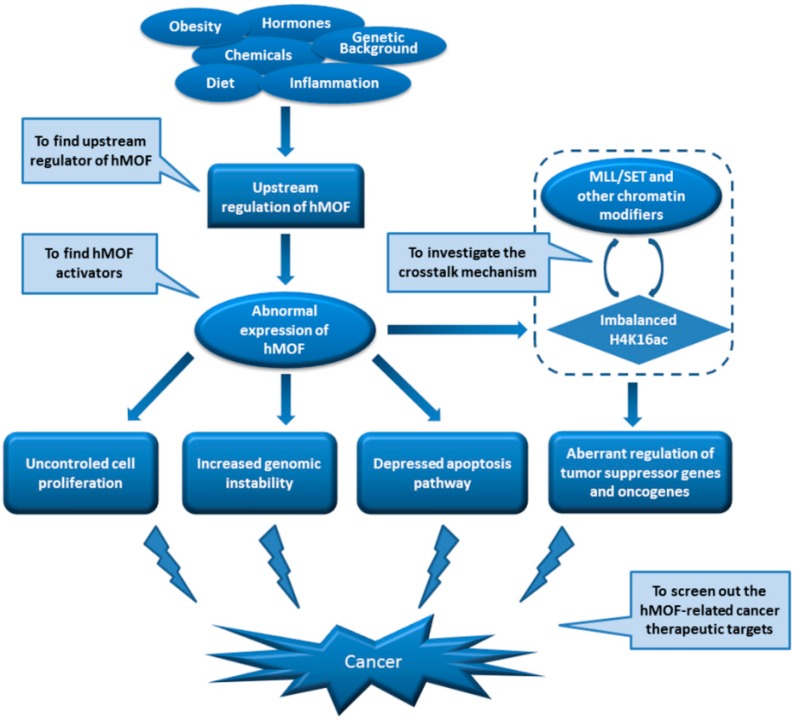
Schematic diagram of human MOF (hMOF)-related mechanisms in the process of tumorigenesis. MLL, mixed-lineage leukemia.

As a member of the MYST family of histone acetyltransferases, MOF is evolutionarily conserved from fly to human. In cells, MOF can form two distinct multiprotein complexes (MSL and NSL). Both complexes can acetylate histone H4K16. The NSL complex can also acetylate histone H4 at lysines 5 and 8. Research evidence has demonstrated that MOF functions in genomic instability, spontaneous chromosomal aberrations, cell cycle defects, reduced transcription of certain genes, defective DNA damage repair and early embryonic lethality. However, except for the different cellular outputs directed by MOF, the detailed signal transduction pathway linking upstream stimuli to MOF activity was so far poorly understood, as well as the distinct regulation territories ruled by MSL and NSL complexes, respectively. Further investigations into these questions will help us better understand how MOF acts in tumorigenesis and what we can do to utilize MOF-associated pathways in cancer prevention and treatment. Interestingly, abnormal gene expression of human MOF and its corresponding acetylation of H4K16 have been found in certain types of primary cancer tissues, including breast cancer, medulloblastoma, ovarian cancer, renal cell carcinoma, colorectal carcinoma, gastric cancer, as well as non-small cell lung cancer. Although the involvement of MOF in a variety of cancer cells has been identified, further research is needed to identify the relationship between the MOF expression and different types of cancer prognosis. A schematic diagram showing the MOF-related mechanisms in the process of tumorigenesis is represented in Figure 4.

## Abbreviations

MYST, Moz-Ybf2/Sas3-Sas2-Tip60; HATs, histone acetyltransferases; H4K16, histone H4 at lysine 16; HDACs, histone deacetylases; MOF, males absent on the first; MSL, male-specific lethal; H4K16ac, H4K16 acetylation; DCC, dosage compensation complex; WDR5, WD repeat domain 5; NSL, non-specific-lethal; H3K4me2, histone H3K4 di-methylation; DDR, DNA damage response; DSBs, double-strand breaks; HR, homologous recombination; NHEJ, nonhomologous end-joining; ATM, ataxia-telangiectasia-mutated; HDACi, HDAC inhibitors.
